# Quantitative spatial analysis of crystallin proteins in human lens epithelial cells

**DOI:** 10.1038/s41598-025-17896-0

**Published:** 2025-10-03

**Authors:** Alessandro Cristoforetti, Giorgio Baldessari, Lizaveta Chychko, Ignacio Babiloni Chust, Samuele Sartori, Sonja Schickhardt, Flavia Ravelli, Silvia Bertoluzza, Matthias Carl, Saadettin Sel, Gerd U. Auffarth, Lucia Poggi

**Affiliations:** 1 Department of Cellular, Computational and Integrative Biology – CIBIO, University of Trento, Trento, 38123 Italy; 2 Department of Ophthalmology, Heidelberg University, Heidelberg, 69120 Germany; 3 CISMed – Centre for Medical Sciences, University of Trento, Trento, 38123 Italy; 4 Istituto di Matematica Applicata e Tecnologie Informatiche, CNR, Pavia, 27100 Italy

**Keywords:** Biotechnology, Cell biology, Molecular biology, Biomarkers, Molecular medicine, Diseases, Eye diseases, Biological techniques, Biological models, Cytological techniques, Gene expression analysis, Imaging

## Abstract

Resolving spatial protein dynamics in native human epithelial tissues presents a significant technical challenge, particularly in inherently curved or unevenly mounted specimens. Here, we introduce an image processing pipeline for high-resolution, compartment-based analysis of protein localization, using the native three-dimensional architecture of the human anterior lens epithelium and capsule complex as a robust ex vivo proof-of-principle platform for precise cell segmentation and quantitative analysis. This platform integrates whole-mount immunostaining, 3D confocal imaging, computational tissue flattening, digital segmentation, and spatial regression to quantitatively map subcellular protein distributions at the tissue scale. To demonstrate the utility of this approach, we examined the spatial distribution of αB-crystallin (CRYAB), a stress-associated small heat shock protein, and βB2-crystallin (CRYBB2), a predominantly structural lens protein, in specimens obtained during cataract surgery. We observed a marked accumulation of CRYAB in epithelial cells at the capsule edge following both laser and manual capsulorhexis, indicating a localized stress response to surgical intervention. In contrast, CRYBB2 distribution remained unaffected. Furthermore, both proteins exhibited consistent cytoplasmic localization, while only CRYBB2 occasionally showed exclusive nuclear accumulation. This pipeline offers a scalable framework for quantitatively resolving protein localization in native epithelial architectures, using CRYAB and CRYBB2 as examples of how stress-associated changes can be spatially mapped in situ within the human lens.

**Supplementary Information:**

The online version contains supplementary material available at 10.1038/s41598-025-17896-0.

## Introduction

The eye offers a unique opportunity for studying systemic and ocular health through its accessible tissues, such as the retina, vasculature, and lens. These tissues allow for measuring molecular and cellular biomarkers that reflect ageing and disease states^1–6^.

Among these, the lens stands out due to its morphological simplicity and accessibility, making it a prime focus for research on epithelial development and function, ageing, regeneration and disease^7–11^. The lens consists of two main subcellular compartments encased in a collagen-rich basal membrane called the lens capsule^12^. The center contains terminally differentiated fiber cells that lack organelles and are rich in crystallins, maintaining transparency and refractive function. The anterior hemisphere is covered by a monolayer of highly polarized lens epithelial cells (LECs), with their basal surfaces attached to the capsule and apical sides facing the fiber cells^13,14^.

LECs are metabolically active and vital for capsule production, nutrient support, and long-term lens homeostasis^8^. Oxidative stress, inflammation, medications, ageing, and systemic conditions can impair LEC function, potentially leading to cataract formation, the world’s leading cause of blindness^10,15–17^. Also, LEC dysfunction may influence neighbouring ocular structures such as the retina and vasculature. Because of their central role in maintaining lens and retinal health, stress response in LECs and their behavior is a focus of research interest^18–21^.

Cataract surgery, involving manual or laser-assisted capsulorhexis and lens fiber removal, leaves behind residual LEC. These cells may engage in abnormal wound-healing responses or lens regeneration, potentially leading to epithelial-mesenchymal transition, posterior capsular opacification (PCO), and further complications affecting ocular tissues^22–25^. The frequent occurrence of such complications has prompted studies comparing conventional and femtosecond laser-assisted cataract surgery (FLACS) regarding stress activation in LECs^26–32^. Thus, LECs play central roles in maintaining lens and retinal health, and assessing their stress responses has become an important focus of research.

Crystallin proteins, particularly the α-crystallins (αA and αB), are emerging as key stress-responsive proteins with potential as biomarkers and therapeutic targets^33–35^. As members of the small heat shock protein family, they contribute to cellular homeostasis by preventing protein aggregation under stress^36,37^. Among them, αB-crystallin (CRYAB) is notably upregulated in response to a wide range of physiological and pathological stressors, can be secreted via exosomes, and has been implicated in neuroprotective and anti-inflammatory processes^34,38–44^. Within the lens, recent studies have identified a more dynamic role for CRYAB in LECs following cataract surgery, where its expression rapidly shifts in coordination with inflammatory and fibrotic responses^24,45–47^. In contrast, βB2-crystallin (CRYBB2), a member of the β/γ-crystallin superfamily, has traditionally been regarded as a structural component of the lens^48^. However, emerging evidence suggests that CRYBB2 may also participate in stress-related functions beyond the lens, including roles in neuroprotection and brain physiology^49–53^. Together, these findings underscore the relevance of crystallins, particularly CRYAB, as early markers of cellular stress, warranting further investigation in the context of surgical injury to the lens epithelium.

To address the technical challenge of analyzing protein distribution across inherently curved and variably mounted epithelial tissues, we developed a computational imaging pipeline that integrates tissue flattening, 3D immunofluorescence quantification, and compartment-based single-cell segmentation across the full anterior lens epithelium. As a proof-of-principle application, we implemented this pipeline on human anterior lens capsule cells (ALCCs) obtained shortly after cataract surgery. We selected CRYAB, a stress-inducible molecular chaperone, and CRYBB2, a predominantly structural lens protein, as representative markers to evaluate the platform’s capacity for resolving spatial and subcellular protein distribution patterns. This approach enables spatially resolved, quantitative analysis of protein localization within intact epithelial architectures and provides a scalable framework for investigating dynamic cellular responses, such as those triggered by surgical injury, in the human lens and other tissue contexts.

## Results

### Implementing a digital image processing pipeline for 3D confocal analysis of protein distribution in ALCC

To investigate whether CRYAB protein distribution in LEC reflects a localized stress response, we performed whole-mount immunohistochemistry on ALCC using an anti-CRYAB antibody. To assess the specificity of CRYAB behavior, we also applied the same workflow to CRYBB2, another crystallin protein. The ALCC were then flat-mounted and imaged using 3D confocal microscopy, producing 63 multichannel image stacks representing DAPI, CRYAB, and CRYBB2 fluorescent signals and a bright field covering both the capsulotomy cut and the interior bulk of LEC.

**Fig. 1:**
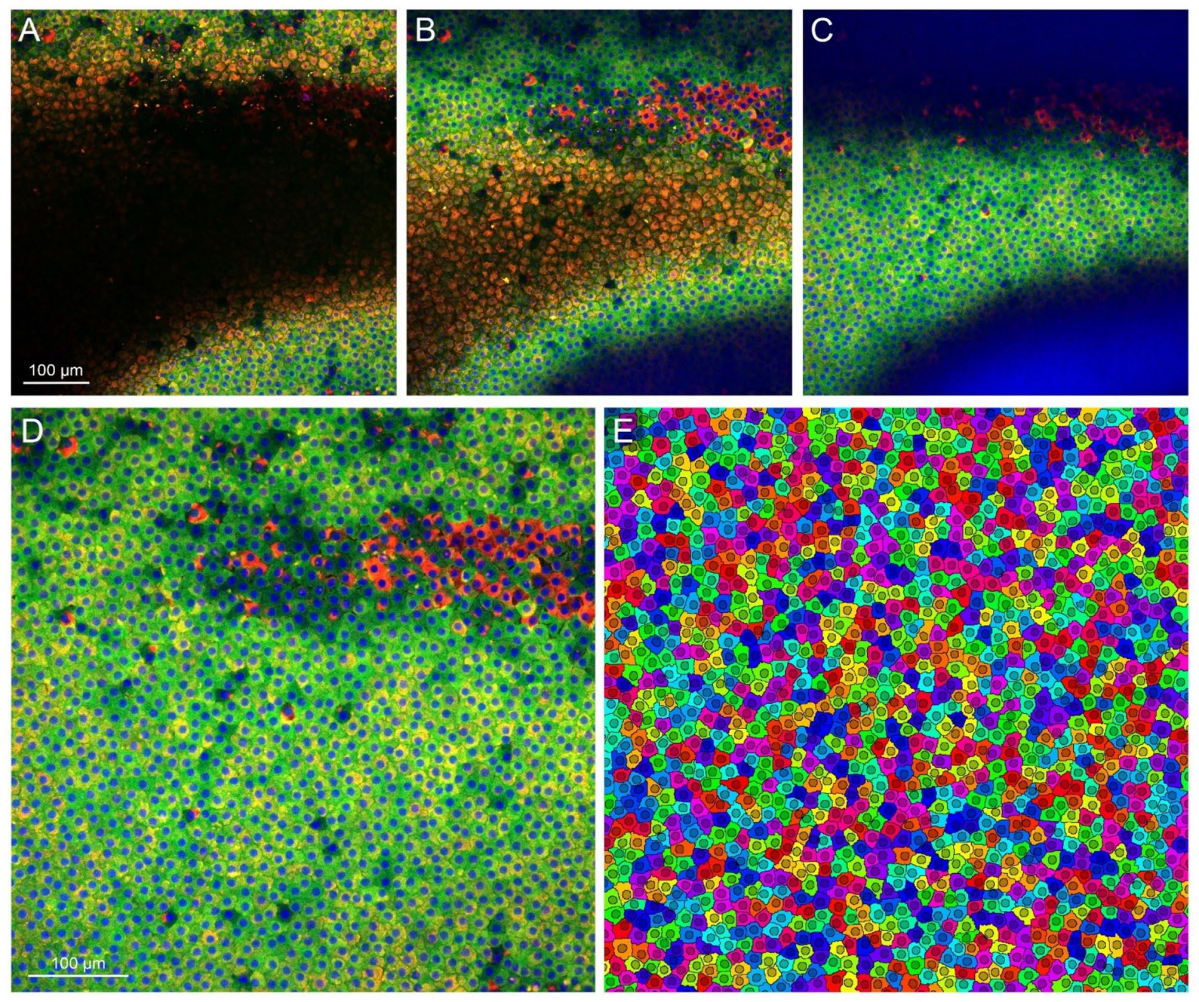
Focal correction and compartmental segmentation of an exemplificative ALCC microscopy image stack. The CRYAB, CRYBB2, and DAPI signals in the interior of LEC are represented in the red, green, and blue channels of the RGB image, respectively, with contrast adjusted between 0 and 99 percentiles. **A** – **C**) Slices 3, 8, and 13 of the original stack. **D**) Final full field in-focus image obtained from focal correction of the image stack. **E**) Colorized label image resulting from the compartmental segmentation of the in-focus image. Black pixels represent compartment borders, the cytoplasm of each cell is represented by a different color, the nucleoplasm by a darker version of the same color.

Given that mounted ALCC samples often exhibit uneven surfaces, resulting in a partially usable image field in each slice (Fig. 1A–C), we implemented a digital image processing pipeline to improve quantitative reliability. Specifically, focal correction via inter-slice interpolation was applied across the DAPI (nuclear), CRYAB, and CRYBB2 channels to generate sharp, in-focus images from each confocal z-stack (Fig. 1D). This approach allows for accurate quantification of protein signal intensity across the tissue, from the capsulotomy edge to the central region (see Materials and Methods).

To analyze CRYAB and CRYBB2 localization within the tissue, both in relation to one another and across cellular compartments, we performed automatic segmentation of the in-focus images using a marker-controlled watershed algorithm (Fig. 1E), applied to the DAPI signal and cellular morphological features. This enabled the separate detection of each cell’s cytoplasm and nucleoplasm, allowing for compartment-specific quantification of CRYAB and CRYBB2 fluorescence intensity. This analysis facilitated correlation assessment between CRYAB and CRYBB2 expression in the nucleus and cytoplasm, providing insights into their potential colocalization or independent regulation within LECs.

### Capsulorhexis induces a specific early accumulation of CRYAB, but not CRYBB2, at the LEC border

**Fig. 2:**
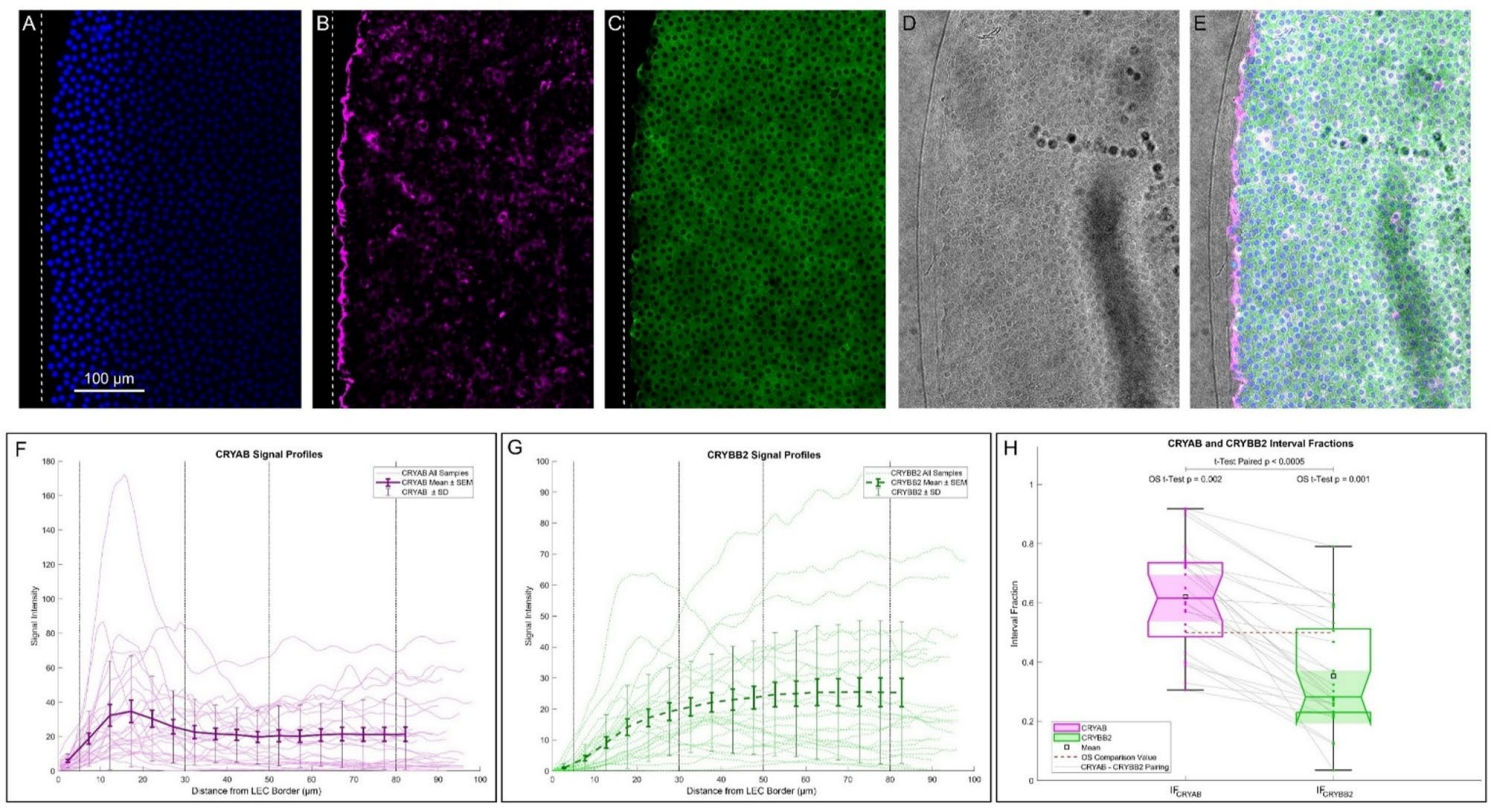
Analysis of CRYAB and CRYBB2 signals in relation to capsulotomy distance. **A** – **C**) Signal of DAPI (**A**), CRYAB (**B**), and CRYBB2 (**C**) in the ALCC region proximal to capsulotomy in a sample. The dotted line defines the position of the LEC border. **D**) Bright field of the ALCC image. **E**) Composite image of **A** – **D**. **F**) Vertical average of CRYAB signal as function of distance from LEC border for all the samples with mean (bold line), SD (bars), and SEM (bold bars). The vertical dotted lines define the proximal (5–30 μm) and distal (50–80 μm) intervals from LEC border. G) Vertical average of CRYBB signal as function of distance from LEC border for all the samples with mean (bold line), SD (bars), and SEM (bold bars). **H**) Box plot of interval fractions for CRYAB and CRYBB2 (OS) comparison with the equidistributional value (0.5) and paired comparison between proteins. Values above 0.5 indicate a predominance of signal in the proximal interval.

To investigate the spatial distribution of CRYAB and CRYBB2 protein signals relative to the LEC border on the capsulotomy side, we analyzed 25 samples. Each image was rotated to align the surgical cut vertically (example in Fig. 2A–E), and the vertical average of the fluorescence signals was calculated. This vertical average was then aligned horizontally with the LEC border (Fig. 2A–C, dotted line) and expressed as a function of metric distance for CRYAB (Fig. 2F) and CRYBB2 (Fig. 2G). The mean, standard deviation (SD), and standard error of the mean (SEM) across samples were computed and plotted as a function of distance.

Analysis of the CRYAB curve (Fig. 2F) revealed that the signal intensity peaks in LEC located immediately adjacent to the capsulotomy edge (proximal region, 5–30 µm) and decreases in more distal regions (50–80 µm). In contrast, CRYBB2 showed either no significant spatial variation or a slight increase in intensity with distance from the cut (Fig. 2G). To quantify these patterns, we calculated the interval fraction (IF), defined as the fraction of each protein’s total signal found in the proximal region relative to the average signal across both proximal and distal intervals. Figure 2H shows the box plots of CRYAB interval fraction $\:{IF}_{CRYAB}^{}$ (mean = 0.621, SD = 0.179, CI_mean_ = 0.551–0.691) and of CRYBB2 interval fraction $\:{IF}_{CRYBB2}^{}$ (mean = 0.353, SD = 0.186, CI_mean_ = 0.281–0.426). A one-sample test against the equidistributional value (0.5) revealed a significant proximal accumulation of CRYAB (OS p = 0.002, Cohen’s d = 0.676) with 76% of the samples showing this trend. Conversely, CRYBB2 exhibits significantly lower proximal values (OS p = 0.001, Cohen’s d = −0.789) with only 28% of the samples presenting proximal accumulation. Pairwise comparison between the two proteins confirmed that $\:{IF}_{CRYAB}^{}$ is significantly higher than $\:{IF}_{CRYBB2}^{}$ (p < 0.0005, Cohen’s d = 1.467, Cohen’s d’ = 1.695) in 100% of the samples. These findings suggest a rapid and localized CRYAB-specific accumulation pattern near the incision site, consistent with an acute LEC stress response triggered by surgical trauma.

### Pixel-wise colocalization analysis detects Spatial correlation of CRYAB and CRYBB2 in the LEC epithelium

Although CRYBB2 did not exhibit spatial redistribution near the capsulotomy edge, unlike CRYAB, previous studies have suggested that the two crystallin proteins may physically interact or be co-regulated under stress conditions^52,54^. This prompted us to explore whether CRYAB and CRYBB2 exhibit spatial colocalization in the lens epithelial cell (LEC) layer under physiological conditions.

**Fig. 3:**
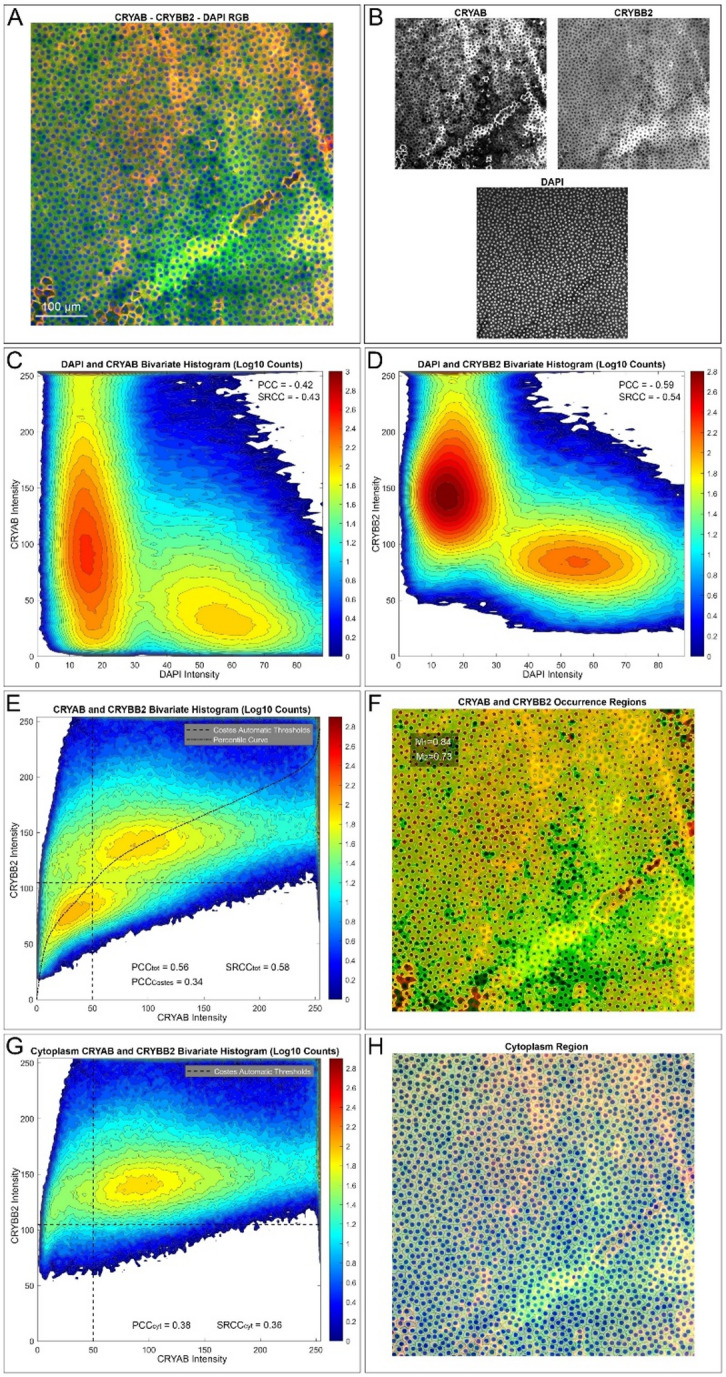
Pixelwise analysis of the in-focus image of an exemplificative sample. **A**) RGB image showing the spatial distribution of CRYAB (red channel), CRYBB2 (green channel), and DAPI (blue channel) signals. **B**) The image channels shown separately. C and **D**) Bivariate count histograms of DAPI signal versus CRYAB and CRYBB2, respectively, in base 10 logarithmic scale. The Pearson’s correlation coefficients (PCC) and Spearman’s rank correlation coefficient (SRCC) are reported. **E**) Bivariate count histogram of CRYAB versus CRYBB2 signals with superimposed percentile curve and Costes automatic thresholds. PCC and SRCC of the whole histogram, PCC_tot_ and SRCC_tot_, respectively, and PCC of the Costes cooccurrence region (PCC_Costes_) are reported. **F**) CRYAB and CRYBB2 co-occurrence region based on Costes thresholds is visualised as overlays over the image. The CRYAB exclusive occurrence region is red, the CRYBB2 exclusive occurrence region is green, and the CRYAB–CRYBB2 co-occurrence region is yellow. Mandes coefficients M_1_ and M_2_ based on the cooccurrence region are reported as well. **G**) Bivariate count histogram of CRYAB versus CRYBB2 signals restricted to the segmented cytoplasmatic region with corresponding PCC (PCC_cyt_) and SRCC (SRCC_cyt_). **H**) Segmented cytoplasmatic region shown as white overlay.

Using pixel-wise colocalization techniques on 63 in-focus, interpolated confocal ALCC images (Fig. 3A–B), we assessed the spatial correlation between CRYAB and CRYBB2 signals. Bivariate histograms of pixelwise intensity are an effective method to visualize correlative relationships between images, with Pearson’s correlation coefficient (PCC) measuring their linear relationship and, similarly, Spearman’s rank correlation coefficient (SRCC) measuring the non-linear relationship. Each correlation coefficient was verified by a significance test performed by image randomization (Supplementary Table 1). Bivariate histograms revealed a bimodal distribution relative to DAPI, indicating predominant cytoplasmic localization for both proteins (Fig. 3C–D). Both CRYAB and CRYBB2 were significantly negatively correlated with DAPI (CRYAB: PCC = − 0.22 ± 0.10; CRYBB2: PCC = − 0.31 ± 0.15; *p* < 10⁻²⁴), supporting their exclusion from the nucleus. Direct correlation between CRYAB and CRYBB2 was significantly positive (PCC = 0.54 ± 0.14; SRCC = 0.67 ± 0.11; *p* < 10⁻³⁸), indicating spatial co-expression.

Global correlation may be influenced by shared spatial localization rather than true molecular interaction (resulting in the two peaks of the bivariate histogram affecting the correlation coefficients shown in Fig. 3E). To distinguish genuine colocalization from mere spatial compartment cooccurrence, we used the Costes method^55^. This analysis identified a high-confidence colocalization region in the cytoplasm (Fig. 3E–F), with a reduced but still significant correlation (PCC_Costes_ = 0.41 ± 0.19; *p* < 10⁻²⁴). Manders coefficients (M_1_ = 0.81 ± 0.16; M_2_ = 0.75 ± 0.18) confirmed high colocalization, though lower M2 suggested instances of exclusive CRYBB2 presence. Validation via segmentation-based cytoplasmic masking (Fig. 3G–H) yielded consistent results (PCC_cyt_ = 0.50 ± 0.16; SRCC_cyt_ = 0.60 ± 0.16; *p* < 10⁻³³), demonstrating true spatial correlation of CRYAB and CRYBB2 in the cytoplasm of LEC.

Altogether, this analysis supports the conclusion that CRYAB and CRYBB2 exhibit true spatial correlation in the LEC epithelium, with exclusive occurrence being more frequent for CRYBB2 than CRYAB.

### Compartment-based profiling uncovers predominant cytoplasmic distribution of CRYAB and CRYBB2 and rare, independent nuclear enrichment of CRYBB2 in LEC

**Fig. 4:**
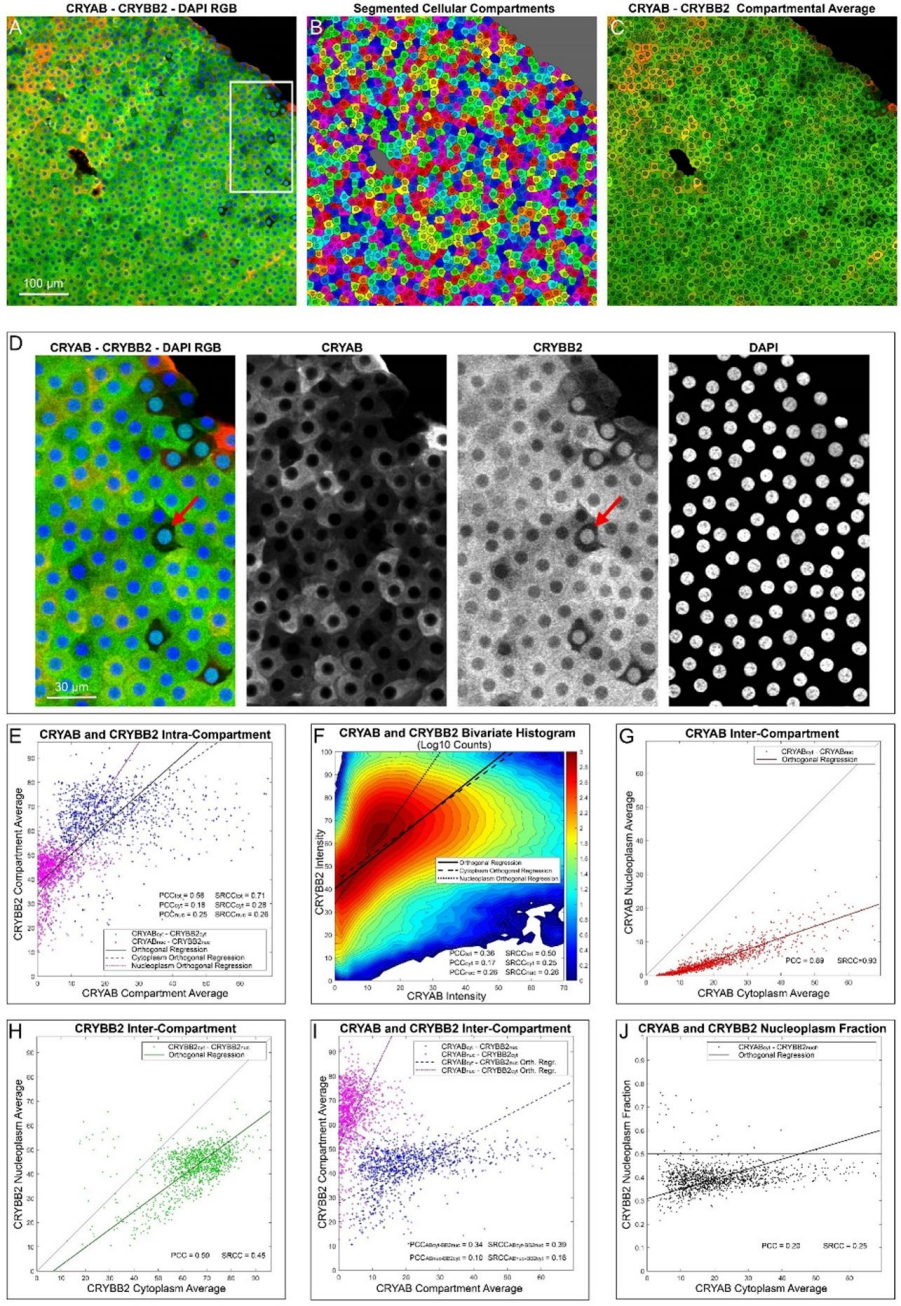
Compartment-based analysis of the in-focus image of an exemplificative sample. **A**) RGB image showing the spatial distribution of CRYAB (red channel), CRYBB2 (green channel), and DAPI (blue channel) signals. **B**) Corresponding compartmental segmentation of the cytoplasm of each cell, the nucleoplasm (darker color) and the cell-free area (grey). **C**) Image of CRYAB and CRYBB2 signal averaged within each cytoplasmic and nuclear compartment, shown in the red and green channels, respectively. **D**) Magnified region of image A (white inset) with channel separation, evidencing nuclei with higher CRYBB2 signal (e.g., red arrow). **E**) Intra-compartment scatterplot of CRYAB versus CRYBB2 averages within the cytoplasm and the nucleoplasm. **F**) Bivariate histogram of the pixelwise CRYAB and CRYBB2 signals of the same image. **G**) Inter-compartment scatterplot of cytoplasm CRYAB versus nucleoplasm CRYAB. **H**) Inter-compartment scatterplot of cytoplasm CRYBB2 versus nucleoplasm CRYBB2. **I**) Inter-compartment scatterplot of cytoplasm CRYAB versus nucleoplasm CRYBB2 and nucleoplasm CRYAB versus cytoplasm CRYBB2. **J**) Scatterplot of CRYAB cytoplasm average versus CRYBB2 nucleoplasm fraction. Orthogonal regressions, PCC, and SRCC are reported in each scatterplot. PCC_tot_ and SRCC_tot_ indicate correlations for all compartments, PCCcyt and SRCC_cyt_ for the cytoplasm, PCC_nuc_ and SRCC_nuc_ for the nuclei, PCCABcyt_−BB2nuc_ and SRCC_ABcyt−BB2nuc_ for cytoplasm CRYAB versus nucleoplasm CRYBB2, PCC_ABnuc−BB2cyt_ and SRCC_ABnuc−BB2cyt_ for nucleoplasm CRYAB versus cytoplasm CRYBB2.

To investigate crystallins localization with greater biological precision, we performed compartment-based profiling on segmented, in-focus ALCC images (Fig. 4A–B). This approach enabled accurate identification of each cell’s cytoplasmic and nucleoplasmic regions, allowing quantification of CRYAB and CRYBB2 signal distribution on a per-cell basis (Fig. 4C). Unlike pixel-wise colocalization, which reflects spatial signal overlap at subcellular resolution, compartment-based analysis provides protein quantification within defined biological units, facilitating detection of subtle patterns, such as atypical nuclear accumulation in individual cells that may be obscured by aggregate spatial statistics (e.g., Fig. 4D).

Average compartmental intensities were used to explore both intra- and inter-compartment relationships between CRYAB and CRYBB2. Intra-compartment correlations were calculated by comparing CRYAB and CRYBB2 intensities in either the cytoplasm or nucleus of each cell (Fig. 4E). These correlations were significantly positive (PCC = 0.65 ± 0.15, SRCC = 0.80 ± 0.08, *p* < 10⁻⁴¹) and exceeded those obtained from pixel-wise colocalization (Fig. 4F), highlighting the value of this method for isolating biologically relevant trends. The correlation remained significant within each compartment type: cytoplasm (PCC = 0.57 ± 0.19, SRCC = 0.69 ± 0.17, *p* < 10⁻³¹) and nucleoplasm (PCC = 0.65 ± 0.16, SRCC = 0.75 ± 0.16, *p* < 10⁻³⁹), indicating that co-expression of CRYAB and CRYBB2 is not simply due to shared cytoplasmic localization.

We next examined inter-compartment relationships by correlating nuclear and cytoplasmic levels of each crystallin within individual LEC (Fig. 4G–H). CRYAB levels in the cytoplasm were strongly associated with those in the nucleus (PCC = 0.81 ± 0.07; SRCC = 0.85 ± 0.09, *p* < 10⁻⁶⁰), and a similar, albeit more variable, pattern was observed for CRYBB2 (PCC = 0.75 ± 0.25; SRCC = 0.83 ± 0.16, *p* < 10⁻³²). Cross-compartment correlations between CRYAB and CRYBB2 also revealed significant associations, particularly between cytoplasmic CRYAB and nuclear CRYBB2 (PCC = 0.65 ± 0.15; SRCC = 0.74 ± 0.14, *p* < 10⁻⁴¹), and to a lesser extent in the reverse direction (PCC = 0.50 ± 0.19; SRCC = 0.62 ± 0.18, *p* < 10⁻²⁸). Thus, compartment-based analysis reveals a strong correlation between the presence of each crystallin in the nucleus and the cytoplasm.

Analysis of inter-compartment relationships revealed strong correlations between nuclear and cytoplasmic expression levels of both CRYAB (PCC = 0.81 ± 0.07, SRCC = 0.85 ± 0.09, *p* < 10⁻⁶⁰; Fig. 4G) and CRYBB2 (PCC = 0.75 ± 0.25, SRCC = 0.83 ± 0.16, *p* < 10⁻³²; Fig. 4H), suggesting coordinated distribution of each protein between compartments. Moreover, inter-compartment correlations were also significant: cytoplasmic CRYAB correlated with nuclear CRYBB2 (PCC = 0.65 ± 0.15, SRCC = 0.74 ± 0.14, *p* < 10⁻⁴¹), while nuclear CRYAB correlated with cytoplasmic CRYBB2 to a lesser extent (PCC = 0.50 ± 0.19, SRCC = 0.62 ± 0.18, *p* < 10⁻²⁸; Fig. 4I). These results are consistent with prior colocalization findings and suggest overlapping, though not identical, regulation patterns.

Despite overall cytoplasmic enrichment, nuclear fractions of CRYAB and CRYBB2 confirmed distinct localization trends. On average, nuclear fractions were modest for both proteins ($\:\langle{CRYAB}_{nucfr}^{}\rangle$ = 0.206 ± 0.085, $\:\langle{CRYBB2}_{nucfr}^{}\rangle$ = 0.263 ± 0.094), confirming their cytoplasmic predominance. However, CRYBB2 was more prone to nuclear accumulation and exhibited higher variability. Interestingly, a minority of cells showed exclusive nuclear localization of CRYBB2 (Fig. 4D), a pattern not observed for CRYAB. Classification of cells into high-nuclear-fraction (HNF) and low-nuclear-fraction (LNF) subgroups revealed that CRYBB2 HNF cells were more frequent (5.09 ± 7.33%) than CRYAB HNF cells (1.94 ± 2.45%), with some samples exceeding 15% CRYBB2 HNF cells. Furthermore, while nearly all CRYAB-positive cytoplasms expressed CRYBB2, the reverse was not true: some CRYBB2-positive cytoplasm lacked detectable CRYAB (Fig. 4C and E), like in the earlier Manders analysis (M_2_ < M_1_).

To investigate the potential relationship between cytoplasmic CRYAB and nuclear CRYBB2, we quantified the correlation between cytoplasmic CRYAB intensity and CRYBB2 nuclear fraction (Fig. 4J). A significant positive association emerged (PCC = 0.46 ± 0.18, SRCC = 0.58 ± 0.21, *p* < 10⁻²⁸), suggesting that high cytoplasmic CRYAB may coincide with increased nuclear CRYBB2 accumulation. However, this trend was not universal. In a focused analysis of samples with ≥ 5 CRYBB2 HNF cells (53/63 samples), 62.2% showed significantly higher cytoplasmic CRYAB in the HNF group (+ 190.4 ± 186.0%, *p* < 0.05), while 13.2% showed the opposite trend (–44.7 ± 25.7%, *p* < 0.05). These divergent patterns may reflect underlying heterogeneity in cell state, regulatory mechanisms, or stress responses.

Altogether, compartment-based profiling reveals robust cytoplasmic coexpression of CRYAB and CRYBB2 in LECs and provides complementary insights beyond pixel-wise colocalization, including the identification of rare CRYBB2 nuclear accumulation events and cell-to-cell variability that may be biologically meaningful.

### Capsulotomy type does not impact CRYAB/CRYBB2 accumulation at the cutting edge despite differential cell loss

**Fig. 5:**
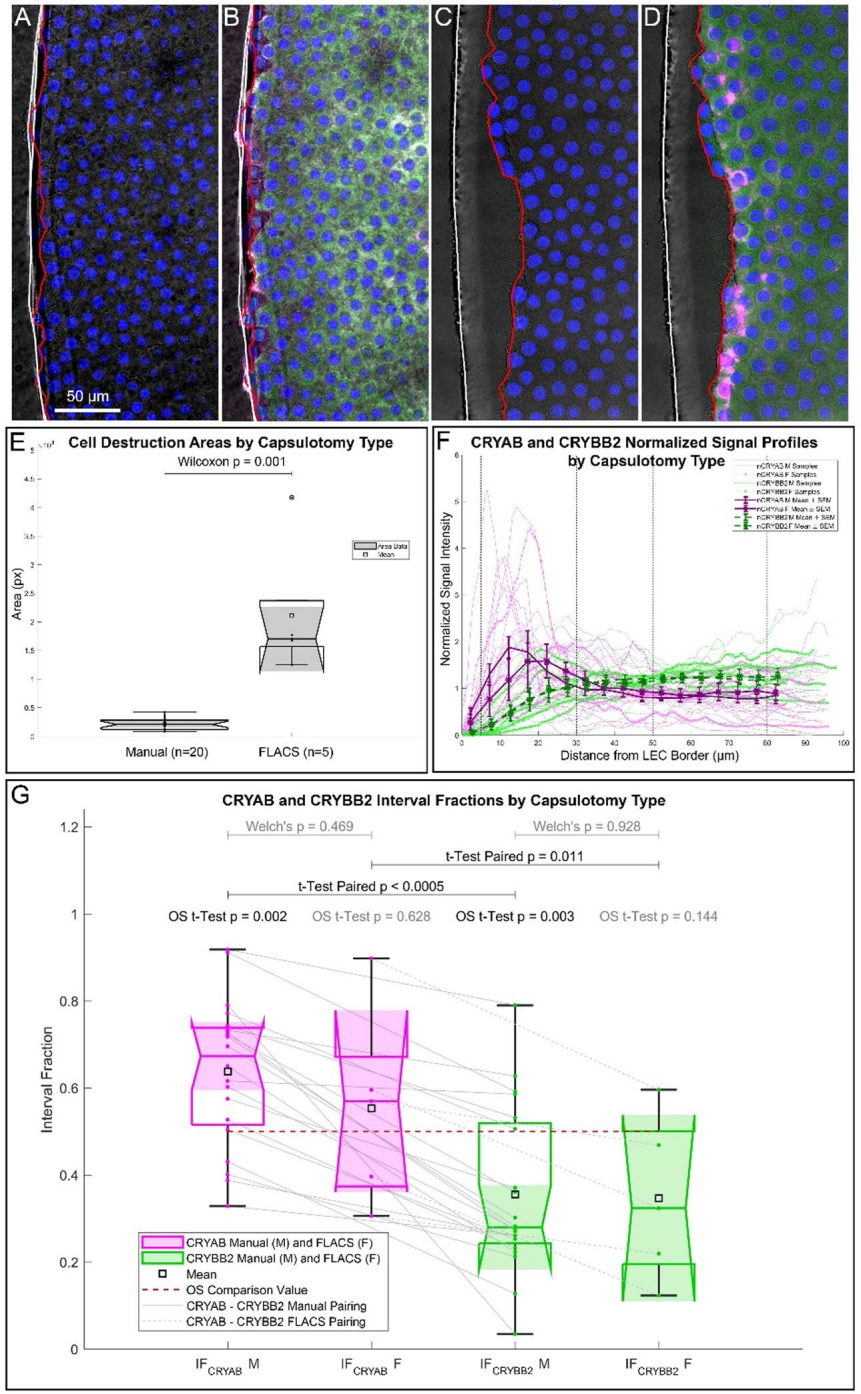
Analysis by capsulotomy type. A – D) Detection of cell destruction areas in a manual capsulotomy sample (**A** and **B**) and a FLACS sample (C and D). Bright field and DAPI (blue) composite images (A and C) and bright field, DAPI, CRYAB (magenta), and CRYBB2 (green) composite images (**B** and **D**) are shown. The cell destruction area is comprised between the capsule cut (white line) and the LEC border (red line). **E**) Box plot of cell destruction areas separated by manual capsulotomy and FLACS samples. **F**) Normalized signal intensity of CRYAB ($\:{nCRYAB}^{i}$) and CRYBB2 ($\:{nCRYBB2}^{i}$) as function of distance from LEC border, separated by manual and FLACS capsulotomy. The signal mean, SD, and SEM over the samples are reported. The vertical dotted lines define the proximal (5–30 microns) and distal (50–80 microns) intervals from LEC border. **G**) Box plot of CRYAB ($\:{IF}_{CRYAB}^{i}$) and CRYBB2 ($\:{IF}_{CRYBB2}^{i}$) interval fractions separated by manual capsulorhexis (M) and FLACS (F) with multiple comparisons between proteins, capsulotomy types, and the equidistributional value 0.5 (significant in black, nonsignificant in grey).

Previous studies reported that femtosecond laser-assisted cataract surgery (FLACS) causes more pronounced cell loss at the anterior capsule margin compared to manual continuous curvilinear capsulorhexis (CCC)^28–30^. To assess whether this differential injury affects local accumulation of CRYAB or CRYBB2, we compared epithelial integrity and crystallin distribution in capsulotomy samples via the two techniques. Cell-free zones were identified in 25 samples (20 CCC, 5 FLACS; see Materials and Methods), with representative examples shown in Fig. 5A–D, and their areas A_CCC_ and A_FLACS_ computed. As expected, FLACS samples exhibited a significantly larger epithelial loss near the incision (Fig. 5E), with A_CCC_ (mean = 2230.3 px^2^SD = 960.5 px^2^CI_mean_ = 1809.3–2651.3 px^2^ smaller than A_FLACS_ (mean = 21147.9 px^2^SD = 11699.5 px^2^CI_mean_ = 10892.8–31402.9 px^2^. Since A_FLACS_ failed the prescribed normality test, non-parametric statistics was also considered for A_CCC_ (median = 2123.4 px^2^, IQI = 1271.6 – 2886.3 px^2^, CI_median_ = 1305.1 – 2825.4 px^2^) and A_FLACS_ (median = 17041.0 px^2^, IQI = 15675.9 – 23721.2 px^2^, CI_median_ = 12500.3 – 41752.9 px^2^), with medians differing significantly according to Wilcoxon p< 0.001 and probability of superiority P(A_CCC_ > A_FLACS_)= 0 %.

We next assessed protein distribution by analyzing normalized CRYAB and CRYBB2 signal intensities both as continuous spatial profiles from the LEC border (Fig. 5F) and as interval fractions separated into the 20 manual ($\:{IF}_{CRYAB}^{}\:M$, $\:{IF}_{CRYBB2}^{}\:M$) and 5 FLACS ($\:{IF}_{CRYAB}^{}\:F$, $\:{IF}_{CRYBB2}^{}\:F$) capsulotomies (Fig. 5G). Confirming the previous results for the whole sample, $\:{IF}_{CRYAB}^{}\:M$ (mean 0.638, SD 0.167, CI_mean_ 0.564–0.711) was significantly larger than $\:{IF}_{CRYBB2}^{}\:M$ (mean 0.355, SD 0.190, CI_mean_ 0.272–0.438) according to a paired T-test (p < 0.0005, Cohen’s d = 1.579, Cohen’s d’ = 1.690). Similarly, $\:{IF}_{CRYAB}^{}\:F$ (mean 0.553, SD 0.227, CI_mean_ 0.354–0.752) was significantly larger than $\:{IF}_{CRYBB2}^{}\:F$ (mean 0.346, SD 0.189, CI_mean_ 0.180–0.512), according to a paired T-test (*p* = 0.011, Cohen’s d = 0.989, Cohen’s d’ = 1.987). However, despite greater epithelial disruption in FLACS samples, neither approach revealed significant differences in local crystallin accumulation compared to CCC. Although CRYAB signal appeared more variable in FLACS cases, the differences between $\:{IF}_{CRYAB}^{}\:M$ and $\:{IF}_{CRYAB}^{}\:F$, and between $\:{IF}_{CRYBB2}^{}\:M$ and $\:{IF}_{CRYBB2}^{}\:F$ were not statistically significant.

## Discussion

This study presents an integrative methodological pipeline combining whole-mount immunostaining, 3D confocal imaging, computational tissue flattening, digital segmentation, and compartment-based quantification to enable high-resolution spatial mapping of protein distribution across the native anterior lens capsule and epithelium (ALCC). This framework addresses a critical technical gap in lens and epithelial biology, allowing for spatially resolved, quantitative analysis of subcellular protein distribution at the tissue scale.

As a proof-of-principle application, we used this approach to investigate the localization patterns of αB-crystallin (CRYAB) and βB2-crystallin (CRYBB2) in human ALCC specimens collected minutes after cataract surgery. CRYAB showed robust and spatially confined enrichment at the capsulotomy edge as early as 5–10 min post-surgery, consistent with its established role as an acute stress-responsive chaperone^38,46,56^. In contrast, CRYBB2 maintained a more uniform distribution, lacking significant enrichment near the surgical edge, in line with its function as a structural lens protein^53^. These findings illustrate the utility of our imaging pipeline for high-resolution, descriptive analyses of protein localization in native human tissue. They also leave open crucial mechanistic questions, namely, whether the observed CRYAB accumulation reflects redistribution from cytoplasmic pools, stress-induced translation from pre-existing mRNA reserves known to exist in LECs, or localized uptake of extracellular protein via injury-induced endocytosis^44,47,57^. These possibilities remain to be tested in future studies.

While we confirmed previously reported greater epithelial disruption following femtosecond laser-assisted capsulotomy (FLACS) relative to manual continuous curvilinear capsulorhexis (CCC)^28–30^we did not detect significant differences in CRYAB or CRYBB2 distribution between surgical methods. This apparent lack of correlation between cell loss and crystallin distribution underscores the importance of spatially resolved analysis and highlights open questions requiring further functional validation. For instance, protein accumulation at the wound edge may occur rapidly and saturate within minutes, independent of surgical method. These and other possibilities merit further investigation into the temporal dynamics and upstream regulators of stress protein localization in these different methodological contexts.

Beyond tissue-level trends, our pipeline also enabled compartment-specific quantification of protein localization within individual LECs across the ALCC. We found that both CRYAB and CRYBB2 were present in cytoplasmic and nucleoplasmic regions; however, CRYBB2 exhibited a significantly higher nuclear fraction. Notably, we identified a distinct subset of LECs showing exclusive nuclear CRYBB2 accumulation. Other studies have reported CRYBB2 localization to the nucleus, cytoplasm, or both^58^. Previous studies in congenital cataract and mouse models have shown that CRYBB2 mutations can alter subcellular distribution, including nuclear retention^48,53,58,59^. This nuclear localization, readily detectable via our subcellular segmentation approach, raises new questions about potential non-structural roles for CRYBB2 in gene regulation or chromatin organization. Our platform now provides a valuable tool to explore these and other possible mechanisms governing crystallin localization under different physiologic or stress conditions.

Using spatial correlation analysis at the single-cell level, we also challenged the hypothesis that nuclear CRYBB2 and cytoplasmic CRYAB showed mutually exclusive distributions. Instead, we found a modest but consistent positive correlation, suggesting that CRYAB accumulation and CRYBB2 nuclear localization may reflect parallel or coordinated stress responses. Interestingly, a rare subset of cells displayed the inverse pattern, suggesting different cellular states. Studies on substantially larger sample sets and incorporating mechanistic insights will be needed to determine the significance of these correlations and to clarify whether they reflect coordinated responses to different types of stress or transitional physiological and pathological states^47,60^.

Collectively, these findings highlight the utility of our integrative approach for mapping protein localization dynamics across biological scales in complex, non-planar epithelial tissues. Applied here in the context of lens epithelial injury following cataract surgery, the method is readily adaptable to a range of tissue types, imaging platforms, and protein targets. Future studies could extend this platform to include additional stress-response markers, such as apoptotic regulators, reactive oxygen species (ROS), endoplasmic reticulum (ER) stress markers, or key signaling pathways such as PI3K/AKT/mTOR. Furthermore, integrating spatial protein quantification with temporal profiling, including exosome-associated CRYAB export and transcriptional responses, could provide deeper insight into the transitions from acute injury to long-term remodeling and regeneration.

By resolving spatial protein distribution with subcellular precision in whole-mount native tissue, we believe that this method represents a significant advance for the field of lens biology and offers a broadly applicable tool for studying injury, regeneration, and epithelial plasticity in situ.

## Methods

### Collection and imaging of anterior lens capsule samples

Samples of anterior lens capsules (ALCCs) (*n* = 26) were collected during routine manual continuous curvilinear capsulorhexis (CCC, *n* = 21) and femtosecond laser-assisted cataract surgery (FLACS, *n* = 5) using the LenSx^®^ Femtosecond Laser system (Alcon, USA), all performed by a single, experienced surgeon. Inclusion criteria specified that participants must have age-related cataracts, with no restrictions on age or gender. Patients with a history of prior intraocular surgery, uveitis, vasculitis, proliferative vitreoretinopathy, or neovascular age-related macular degeneration were excluded from participation. Before surgery, written informed consent was obtained from all patients. The study protocol received approval from the local Ethics Committee of the University of Heidelberg and was conducted by the Declaration of Helsinki. All investigations followed protocols approved by the University of Heidelberg Ethics Committee.

### Immunohistochemistry *(IHC)*

At the start of each surgery, ALCCs were collected and placed in formalin within 5 min during the operation, directly after the procedure. The samples were fixed in formalin for 30 min and then stored at 4 °C until analysis.

The samples were transferred to a well-plate and washed with PBST (1X PBS, 0.1% Triton X-100) and incubated in blocking solution (0.1% Triton X-100 and NGS 10% in 1X PBS) for 2 h at room temperature (RT). Each primary antibody was diluted in blocking solution at a concentration of 1:500 and incubated at 4 °C for 48 h in a humidified chamber. After washing the primary antibody several times with PBST, the samples were incubated with the fluorescent secondary antibody, diluted 1:1000 in blocking solution for 48 h at 4 °C in a humidified chamber. Finally, the samples were mounted on a slide using the mounting medium VectaShield with DAPI (Vector Laboratories, Burlingame, CA) 48 h before the image acquisition. The following antibodies were used:

**Table:** 

Antibody	Company	Reference
CRYBB2	Merck	HPA043749
CRYAB	Abcam	Ab13496
Goat Alexa Fluor 488 anti-Rabbit	Invitrogen	A11034
Goat Alexa Fluor 568 anti-Mouse	Invitrogen	A11004

### Image acquisition

Images were acquired using a Leica SP8 confocal microscope through LAS AF Software 2.6.0 with HC PL FLUOTAR 10×/0.30, HC PL APO 20x/0.5, HC PL APO 40×/1.30 Oil CS2. For every sample at least two different image stacks were taken (between 29 and 55 slices per sample), the first in the inmost part of the lens and the second near the exterior cut. A total of 63 stacks (30 internal and 33 external) were produced. For each sample, 4 channels were acquired: CRYAB in (576–727 nm), DAPI in C2 (410–477 nm), CRYBB2 in C3 (502–548 nm) and brightfield in C4. The image resolution was 1024 × 1024 pixels. Image stacks were opened and processed using Fiji ImageJ software and custom-made functions programmed in MATLAB (MathWorks, Natick, MA).

### Image processing

The 3D stack underwent focal correction to obtain a highly resolved 2D image of the cellular matrix, which was segmented into distinct cells and subcellular compartments (cytoplasm and nucleoplasm).

### Focal correction

**Fig. 6:**
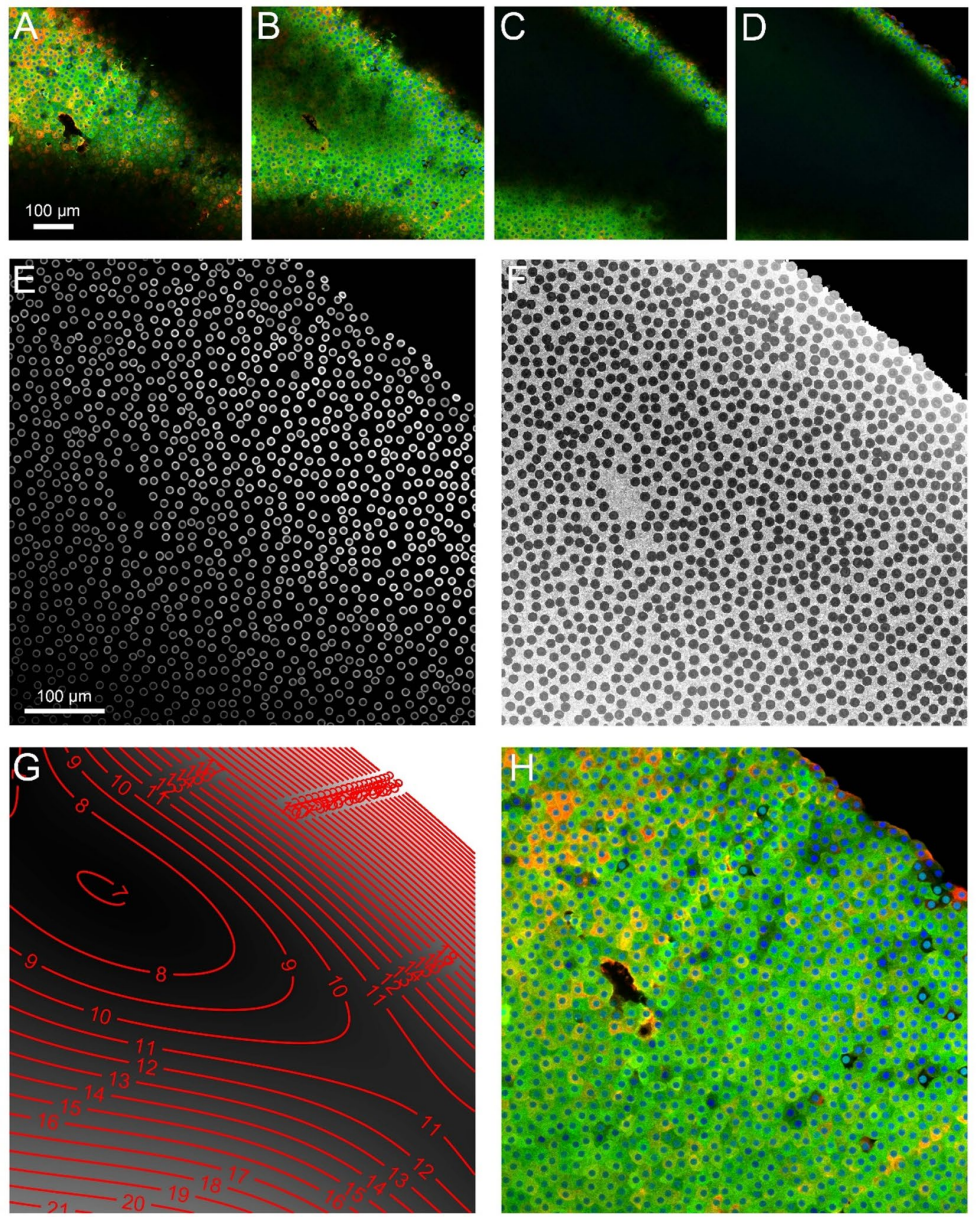
Focal correction in an exemplative 3D stack. **A** – **D**) Sequence of images at different depths in the stack (slice indices = 7, 13, 20, 26), shown as RGB images (red channel = CRYAB, green channel = CRYBB2, blue channel = DAPI). **E**) Maximum DAPI gradient magnitude along the slices (axial direction). **F**) Slice indices corresponding to the maximum. **G**) In-focus manifold represented as isolines of slice indices. **H**) Final in-focus RGB image of the cellular matrix (CRYAB, CRYBB2, DAPI).

We defined $\:x$ and $\:y$ as the planar coordinates in each 2D slice, while $\:z$ was the coordinate that position each slice axially in the acquired 3D stack $\:I\left(x,y,z\right)$, where $\:I$ could indicate the CRYAB, CRYBB2, or DAPI signal. Because of the lack of flatness of the samples, each slice at fixed $\:z$ presented a limited portion of in-focus area, as shown for an exemplative case in Fig. 6A – D. To optimize the analysis, an axial focal correction (along $\:z$, z-correction) was performed, producing a single 2D highly resolved image $\:{I}_{f}\left(x,y\right)$ of the cellular matrix from the whole 3D stack. The z-correction consisted in interpolating the target values on the in-focus surface, a two dimensional manifold passing in the 3D stack expressible in explicit form as $\:{z}_{f}\left(x,y\right)$, representing for each planar position at $\:x$ and $\:y$, the corresponding axial position $\:{z}_{f}$ where the sample image was well focused. The DAPI images, delineating the nuclei, were considered as reference for resolution quality. A 2D Sobel based gradient was computed for each DAPI slice of stack, obtaining the DAPI gradient magnitude $\:{G}_{DAPI}\left(x,y,z\right)$. The maximum of $\:{G}_{DAPI}$ along $\:z$ for each $\:x$, $\:y$ position (Fig. 6E) was computed:

1 $$\:{M}_{DAPI}\left(x,y\right)={G}_{DAPI}\left(x,y,z\right)\equiv\:{G}_{DAPI}\left(x,y,{z}_{m}\right)\:$$

where $\:{z}_{m}\left(x,y\right)$ was the slice coordinate corresponding to the maximum (Fig. 6F), i.e. the slice at a position where the nuclear border was sharpest, if present. Since the nuclear border cover only a small fraction of cellular matrix, $\:{z}_{m}\left(x,y\right)$ was not suitable to be used directly as in-focus surface and needed to be regularized. To exclude low gradient values likely caused by noise instead of actual nuclear borders, all the values of $\:{M}_{DAPI}$ inferior to an automatic threshold were set to zero, according to

2 $$\:{M}_{DAPI}^{th}\left(x,y\right)={M}_{DAPI}\setminus\:\left({M}_{DAPI}<0.1\cdot\:{prc}_{99}\left({M}_{DAPI}\right)\right)$$

where $\:{prc}_{99}$ was the 99 percentiles of $\:{M}_{DAPI}$ and $\:\setminus\:$ denoted the exclusion operator. The in-focus manifold $\:{z}_{f}\left(x,y\right)$ (Fig. 6G) was obtained by fitting an order 5 polynomial on $\:{z}_{m}\left(x,y\right)$ using the least square method weighted by $\:{M}_{DAPI}^{th}$. Based on empirical application on our data, order 5 was deemed a good compromise between manifold regularity and flexibility to adapt to sample unevenness. The final in focus image $\:{I}_{f}$ (Fig. 6H) was obtainined by interpolating $\:I\left(x,y,z\right)$ on $\:{z}_{f}\left(x,y\right)$:

3 $$\:{I}_{f}\left(x,y\right)=I\left(x,y,{z}_{f}\left(x,y\right)\right)$$

Linear interpolation along $\:z$ was used. The interpolation was applied to obtain $\:{CRYAB}_{f}$, $\:{CRYBB2}_{f}$, and $\:{DAPI}_{f}$.

### Image segmentation

**Fig. 7:**
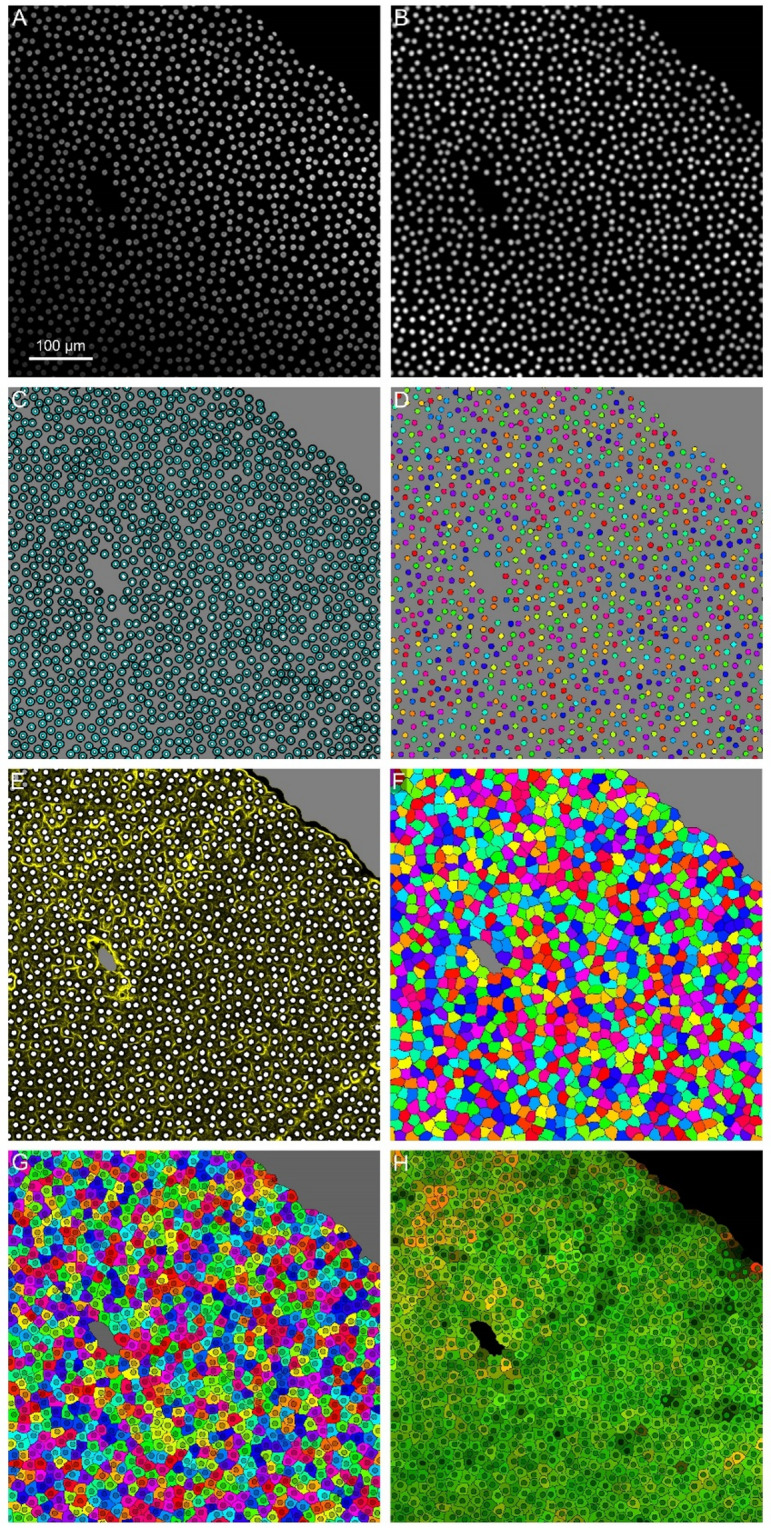
Segmentation procedure of the in-focus image of Fig. 6. **A**) In-focus DAPI signal. **B**) Contrast adjusted DAPI signal. **C**) Input of the first watershed segmentation: nuclear markers (white), extranuclear marker (grey), DAPI gradient (cyan). **D**) Output of the first watershed: inner nuclear space (color labelled) and non-nuclear space (grey). **E**) Input of the second watershed: cellular markers (white), acellular marker (grey), and sum of CRYAB and CRYBB2 gradient magnitudes (yellow). **F**) Output of the second watershed: cells (color labeled) and acellular space (grey). **G**) Final labeled compartmental segmentation: cytoplasmic compartments (light colors), corresponding nuclei (darker color), and acellular space (grey). **H**) RGB image of CRYAB (red channel) and CRYBB2 (green channel) values averaged within cytoplasmic and nuclear compartments.

The segmentation of the cellular compartments was based on the application of two marker-controlled watershed transforms to the gradients of the in-focus images with appropriate binary masks as markers. Its steps for an exemplative case are shown in Fig. 7.

The in-focus DAPI image (Fig. 7A) underwent a local contrast adjustment (LCA) to uniform the contrast between the background and the nuclei intensity over the image. First, a soft background signal $\:{DAPI}_{back}$ was detected from DAPI by applying in sequence Gaussian smoothing, greyscale erosion, dilation, and smoothing again as to remove all the objects under a radius of $\:{r}_{n}$:

4 $$\:{DAPI}_{smooth}=K\left({\sigma\:}_{n}\right)*{DAPI}_{f}$$

5 $$\:DAP{I_{back}} = K\left( {{r_n}} \right)*\left( {\left( {DAP{I_{smooth}} \ominus \:D\left( {{r_n}} \right)} \right) \oplus D\left( {2{r_n}} \right)} \right)$$

where $\:K\left(\sigma\:\right)$ denotes a Gaussian kernel of standard deviation $\:\sigma\:$ pixels, $\:*$ the convolution operator, $\:D\left(r\right)$ a disklike structuring element of radius $\:r$ pixels, $\:\ominus\:$ the grayscale erosion operator, and $\:\oplus$ the dilation operator. Based on our specific dataset, we empirically set $\:{\sigma\:}_{n}$=3 px and $\:{r}_{n}$=9 px. $\:{DAPI}_{back}$ was then subtracted from $\:{DAPI}_{smooth}$ to obtain a soft image containing only the nuclei:

6 $$\:{DAPI}_{nuc}=max\left({DAPI}_{smooth}-{DAPI}_{back},0\right)$$

The unevenness in intensity of $\:{DAPI}_{nuc}$was corrected by applying an automatic local image contrast adjustment^61^. A smooth local nuclear signal intensity $\:{DF}_{nuc}\:$was obtained by fitting an order 2 polynomial on $\:{DAPI}_{nuc}$ (least squares fitting) using $\:{DAPI}_{nuc}$ as a weight. The contrast-adjusted DAPI (Fig. 7B) was computed as:

7 $$\:{DAPI}_{LCA}=\frac{{DAPI}_{nuc}}{{F}_{nuc}}$$

The first watershed transform aimed at identifying the inner nuclear space and was applied to the gradient magnitude of $\:{DAPI}_{f}$, $\:{G}_{{DAPI}_{f}}$ (Fig. 7C, cyan), which was suitable for delineating the nuclear borders. The transform was under the control of nuclear markers $\:{B}_{nuc}$ (Fig. 7C, white) and a marker for extranuclear space $\:{B}_{exnuc}$ (Fig. 7C, grey).

The binary marker mask for the nuclei $\:{B}_{nuc}$ was obtained by robustly detecting the centers of distinct nuclei, i.e., applying an automatically tuned h-maxima transform to the upper 50 percentile of $\:{DAPI}_{LCA}$:

8 $$\:{B}_{nuc}={HMAX}_{h}\left(max\left({DAPI}_{LCA}-{prc}_{50}\left({DAPI}_{LCA}\right),0\right)\right)$$

9 $$\:h=0.1\cdot\:{prc}_{99.9}\left({DAPI}_{LCA}\right)$$

$\:{prc}_{50}$ and $\:{prc}_{99.9}$ indicating the 50 and 99.9 percentiles, respectively.

The binary marker mask for the space outside the nuclei was detected applying a threshold and an erosion to $\:{DAPI}_{LCA}$:

10 $$\:{B}_{exnuc}=\left({DAPI}_{LCA}\le\:{prc}_{50}\left({DAPI}_{LCA}\right)\right)\ominus\:D\left(2\right)$$

The label image $\:{L}_{1}$ resulting from the watershed identified the extranuclear space (label = 1), consisting of cytoplasm and extracellular space, if present, and each distinct nuclear space (label = $\:2\dots\:N+1$) for the $\:N$ nuclei of the image.

The second watershed aimed at separating the distinct cells and was controlled by a marker mask that identified the single cells and consisted in the previously detected nuclear space, i.e., $\:{B}_{cell}={L}_{1}>1$ (Fig. 7E, white), with the addition of a marker for extracellular space $\:{B}_{void}$ (Fig. 7E, grey). The binary marker mask for the area outside the ALCC matrix was obtained as the opening of the thresholded distance transform of $\:{B}_{cell}$:

11 $$\:{B_{void}} = \left( {\left( {dist\left( {{B_{cell}}} \right)> 2 \cdot \:{d_{avg}}} \right) \ominus \:D\left( {{d_{avg}}} \right)} \right) \oplus D\left( {{d_{avg}}} \right)$$

$\:dist$ indicated the Euclidean distance transform and the average internuclear distance $\:{d}_{avg}$ was estimated in correspondence of the maximum of the regularized intensity histogram of $\:dist\left({B}_{cell}\right)$. The second watershed was applied to the sum of the gradient magnitudes of $\:{CRYAB}_{f}$ and $\:{CRYBB2}_{f}$, $\:{G}_{C{RYAB}_{f}}$ and $\:{G}_{{CRYBB2}_{f}}$ respectively, after setting to zero all areas within 2 px from the nuclear space:

12 $${A_2} = \left( {{G_{CRYA{B_f}}} + {G_{CRYBB{2_f}}}} \right) \setminus \:\left( {{B_{cell}} \oplus D\left( 2 \right)} \right)$$

$\:\setminus\:$ was the exclusion operator. $\:{A}_{2}$ (Fig. 7E, yellow) represented the border intensity between cells, based on variation of the target proteins, after killing the interference of the nuclear/cytoplasmic borders. The resulting label image $\:{L}_{2}$ identified the acellular space (label = 1, Fig. 7F in grey) and each cell space (label = $\:2\dots\:N+1$, Fig. 7F multicolor). From the combination of $\:{L}_{1}$ and $\:{L}_{2}$, the nuclear compartments and corresponding cytoplasmatic compartments of all cells were identified and segmented (Fig. 7G).

The average values of $\:{CRYAB}_{f}$ and $\:{CRYBB2}_{f}$ within each nuclear and cytoplasmic compartment was computed (Fig. 7H), obtaining the paired values $\:{CRYAB}_{nuc}^{i}$, $\:{CRYAB}_{cyt}^{i}$, $\:{CRYBB2}_{nuc}^{i}$, $\:{CRYBB2}_{cyt}^{i}$ for the $\:i=1\dots\:N$ cells.

### Analysis of Crystallin distribution in the proximity of the capsulotomy edge

The goal of this analysis is the visualization and study of the distribution of the target proteins in the samples in the capsulorhexis region, more specifically, a restricted ROI area of the image located on the most external region of the lens (Fig. 2, A – E), where the cut is made during the operation. The analysis was performed on 25 image stacks (20 manual and 5 FLACS) with a clear delineation of the capsulotomy region. After rotating the ROI images to grossly align the cut vertically, the profiling function of Fiji ImageJ allowed us to plot the vertical average of the intensity of the two protein channels (CRYAB in magenta, CRYAB2 in green) from the cut to the bulk of the image. The resulting curves (Fig. 2, F and G, dotted lines) were aligned so that the 0 corresponded to the ALCC border, detected at the beginning of cellular expression (Fig. 2, A – C, dashed white line). To investigate different protein accumulation following the trauma of the operation, the *CRYAB*^*i*^ and *CRYBB2*^*i*^ profiles were statistically characterized over the *i = 1…N* samples by the mean, the standard deviation (SD), and the standard error of the mean (SEM). Since the pixel sizes were not equal between samples, the profiles were binned at intervals of 5 micron, and then the statistical descriptors were obtained from the bin averages (Fig. 2, F and G, mean as bold dashed line, SD as bars, SEM as bold bars).

To distinguish between different trends of the target proteins, two typical intervals, one proximal (5–30 micron) and one distal (50–80 micron) from the border region were identified for further analysis (Fig. 2, F and G, vertical dotted black lines). The rationale of this choice is that for distances inferior to 5 micron the profiles show a transient behavior increasing from zero, since the tissue border is irregular, causing the tissue region to come into view gradually. Also, for distances between 30 and 50 microns the profiles reveal a transitional behavior, shifting from proximal to distal region, which could be a confounding factor in the statistics. For distances > 80 microns profile data is not consistently present for all samples, due to the alignment process. Four variables were thus computed for each of the for *i = 1…N* samples: average CRYAB in the proximal and distal intervals ($\:{\langle\:CRYAB\rangle\:}_{\left[5-30\right]}^{i}$ and $\:{\langle\:CRYAB\rangle\:}_{\left[50-80\right]}^{i}$) and average CRYBB2 in the proximal and distal intervals ($\:{\langle\:CRYBB2\rangle\:}_{\left[5-30\right]}^{i}$ and $\:{\langle\:CRYBB2\rangle\:}_{\left[50-80\right]}^{i}$). To appreciate the different distributions between proteins, the behavior of fractional variables was considered, offering the advange of canceling inter-sample intensity variability. Fractions with respect to the whole signal have been preferred over ratios between components to avoid statistical distortions due to the asymptotic behavior for small denominators. The fraction of the signal in the proximal region (interval fraction, IF) was defined on each sample $\:i$ for CRYAB and CRYBB2 ($\:{IF}_{CRYAB}^{i}$ and $\:{IF}_{CRYBB2}^{i}$, respectively) according to the equations:

13 $$\:{IF}_{CRYAB}^{i}=\frac{{\langle\:CRYAB\rangle\:}_{\left[5-30\right]}^{i}}{{\langle\:CRYAB\rangle\:}_{\left[5-30\right]}^{i}+{\langle\:CRYAB\rangle\:}_{\left[50-80\right]}^{i}}$$

14 $$\:{IF}_{CRYBB2}^{i}=\frac{{\langle\:CRYBB2\rangle\:}_{\left[5-30\right]}^{i}}{{\langle\:CRYBB2\rangle\:}_{\left[5-30\right]}^{i}+{\langle\:CRYBB2\rangle\:}_{\left[50-80\right]}^{i}}$$

The IF characterizes how the signal is distributed between the two intervals and the positional trend of proteins. If the IF is 0.5, the protein would be equally distributed, while an IF < 0.5 indicates an accumulation of the protein in the proximal interval.

Paired comparisons of the IFs of the 25 samples between proteins ($\:{IF}_{CRYaB}^{i}$ and $\:{IF}_{CRYbB2}^{i}$) and one sample (OS) comparison toward the uniformity reference value of 0.5 were performed, according to the tests appropriate for the respective populations as explained in Statistical Analysis. In the graphics, interval-based variables for all samples are represented as dot plots superimposed to whiskered box plots (Fig. 2H).

### Pixelwise colocalization analysis

Pixelwise colocalization analysis followed the outline suggested in previous articles^62–64^particularly with respect to the considered metrics and the interpretation of the concepts of colocalization, cooccurrence, and correlation. Bivariate histograms were produced for visual assessment while Pearson’s correlation coefficient (PCC) and Spearman’s rank correlation coefficient (SRCC) quantified the linear and nonlinear relationships between variables. Each correlation coefficient was verified by a significance test performed by image randomization by shuffling 8 × 8 pixels tiles. In the case of CRYAB and CRYBB2 images ($\:{I}_{CRYAB}$ and $\:{I}_{CRYBB2}$), a cooccurrence region $\:{M}_{co}$ for the two signals was identified by applying thresholds $\:{Th}_{CRYAB}$ and $\:{Th}_{CRYBB2}$:

15 $$\:{M}_{co}={I}_{CRYAB}>{Th}_{CRYAB}\cap\:{I}_{CRYBB2}>{Th}_{CRYBB2}$$

The thresholds were automatically obtained based on Costes’ method^55^. In variation with respect to the original method, the relation between the thresholds was set according to a percentile regression instead of an orthogonal (PCA based) regression. This was necessary to address the nonlinearity observed between CRYAB and CRYBB2 signals. In the percentile regression, CRYAB and CRYBB2 values were paired so that they corresponded to the same percentile in both CRYAB and CRYBB2 images. $\:{Th}_{CRYAB}$ and $\:{Th}_{CRYBB2}$ were then detected when the PCC outside the cooccurrence region ($\:{I}_{CRYAB}\le\:{Th}_{CRYAB}\cup\:{I}_{CRYBB2}\le\:{Th}_{CRYBB2}$) reached zero from decreasing values of $\:{Th}_{CRYAB}$ and $\:{Th}_{CRYBB2}$. The cooccurrence was quantified by Manders’ coefficients M_1_ and M_2_^65^computed on the cooccurrence region $\:{M}_{co}$:

16 $$\:{M}_{1}=\frac{\sum\:_{{M}_{co}}{{I}_{CRYAB}}_{i}}{\sum\:{{I}_{CRYAB}}_{i}}$$

17 $$\:{M}_{2}=\frac{\sum\:_{{M}_{co}}{{I}_{CRYBB2}}_{i}}{\sum\:{{I}_{CRYBB2}}_{i}}$$

where $\:{{I}_{CRYAB}}_{i}$ and $\:{{I}_{CRYBB2}}_{i}$ indicate the intensity values at any pixel $\:i$ of images $\:{I}_{CRYAB}$ and $\:{I}_{CRYBB2}$, respectively.

In addition to correlations computed on the whole image and the cooccurrence region, the analysis was also performed separately on the nucleoplasm and cytoplasm areas of the image (without distinction between cells), obtained from the segmentation.

### Compartmental colocalization analysis

The goal of this analysis is to investigate the interrelation of the spatial distribution of the two target proteins with respect to each cell compartment, i.e., cytoplasm and nucleus, in the bulk of the ALCC samples. The average values of the CRYAB and CRYBB2 signals in the cytoplasm ($\:{CRYAB}_{cyt}^{i}$ and $\:{CRYBB2}_{cyt}^{i}$) and nucleoplasm ($\:{CRYAB}_{nuc}^{i}$ and $\:{CRYBB2}_{nuc}^{i}$) of each cell were calculated. The relationships paired in each individual cell were investigated, i.e. between the two crystallins in the same compartment ($\:{CRYAB}_{nuc}^{i}$ vs. $\:{CRYBB2}_{nuc}^{i}$ and $\:{CRYAB}_{cyt}^{i}$ vs. $\:{CRYBB2}_{cyt}^{i}$), in different compartments ($\:{CRYAB}_{nuc}^{i}$ vs. $\:{CRYBB2}_{cyt}^{i}$ and $\:{CRYAB}_{cyt}^{i}$ vs. $\:{CRYBB2}_{nuc}^{i}$), and between the same protein in different compartments ($\:{CRYAB}_{nuc}^{i}$ vs. $\:{CRYAB}_{cyt}^{i}$ and $\:{CRYBB2}_{nuc}^{i}$ vs. $\:{CRYBB2}_{cyt}^{i}$). These relations were visualized in scatterplots and quantified by the computation of the Pearson’s correlation coefficient (PCC) and Spearman’s rank correlation coefficient (SRCC) with p testing for the null hypothesis.

The nucleoplasm fraction of CRYAB and CRYBB2 for each cell were defined as

18 $$\:{CRYAB}_{nucfr}^{i}=\frac{{CRYAB}_{nuc}^{i}}{{CRYAB}_{nuc}^{i}+{CRYAB}_{cyt}^{i}}$$

19 $$\:{CRYBB2}_{nucfr}^{i}=\frac{{CRYBB2}_{nuc}^{i}}{{CRYBB2}_{nuc}^{i}+{CRYBB2}_{cyt}^{i}}$$

The general trend of $\:{CRYBB2}_{nucfr}^{i}$ with respect to $\:{CRYAB}_{cyt}^{i}$ was considered to shed light on how the presence of CRYAB in the cytoplasm correlates with the distribution of CRYBB2 in the cytoplasm and nucleoplasm.

### Analysis by capsulotomy type

A pattern recognition plugin was utilized to measure the space area between the capsule edge and the last cell layer (cell destruction area) to detect differences between manual (M) (as example Fig. 5, A and B) and FLACS (as example Fig. 5, C and D) capsulorhexis. The capsule edge was identified in correspondence with the cut (Fig. 5, A – D, white polyline), while the ALCC border was drawn in correspondence with the cell expression boundary (Fig. 5, A – D, red polyline). Area data were compared between capsulotomy type groups. Area data was compared between capsulotomy type groups (20 and 5 unpaired samples for M and FLACS, respectively), according to the tests appropriate for the respective populations as explained in Statistical Analysis. Results were visualized according to standard whiskered box plot convention (Fig. 5, E).

The protein signal profiles as function of distance were compared between the two capsulotomy types, i.e., manual capsulorhexis (M) and FLACS. To reduce inter-sample variability caused by the acquisition process conditions and increase the detectability of differences, normalized signal profiles were considered. They were obtained dividing each profile by its average value over its domain for all samples:

20 $$\:{nCRYAB}^{i}\left(D\right)=\frac{{CRYAB}^{i}\left(D\right)}{{\langle\:CRYAB\rangle\:}_{\left[0\:80\right]}^{i}}$$

21 $$\:{nCRYBB2}^{i}\left(D\right)=\frac{{CRYBB2}^{i}\left(D\right)}{{\langle\:CRYBB2\rangle\:}_{\left[0\:80\right]}^{i}}$$

The normalized protein profiles $\:{nCRYAB}^{i}$ and $\:{nCRYBB2}^{i}$ were displayed distinguishing between capsulotomy group (Fig. 5, F) and statistically characterized over the samples of each capsulotomy group by the mean, the SD, and the SEM according to the procedure explained previously for the whole population.

The behavior of the IFs of the two proteins, $\:{IF}_{CRYAB}^{i}$ and $\:{IF}_{CRYBB2}^{i}$, was analyzed as well by subdividing the samples into the two subgroups according to capsulotomy types (20 M and 5 FLACS samples). Comparisons were performed OS toward 0.5, intra-group (pairwise), and inter-group (unpaired), according to the tests appropriate for the respective populations as explained in Statistical Analysis. Results were visualized according to standard whiskered box plot convention (Fig. 5, G).

### Statistical analysis

The variables used to describe the samples, i.e. IF and cell destruction area, were described reporting the mean, the standard deviation (SD), and the 95% confidence interval for the mean CI_mean_. Given the low sample size, each population was formally tested for normality with the Shapiro-Wilk test at significance level 0.05, since it has the best statistical power^66^. If not normal, the median, the interquartile interval (IQI), the 95% confidence interval of the median (CI_median_) was reported. The variables analyzed by testing for significant difference between the considered groups. Where the statistical samples in the two groups were paired because represented different measurements on the same physical sample, such as $\:{IF}_{CRYAB}^{i}$ and $\:{IF}_{CRYBB2}^{i}$ for all the 25 physical samples or for the 20 CCC and 5 FLACS separately, a paired comparison was performed. Where this was not possible, because the groups consisted of different physical samples, such as cell destruction areas between CCC and FLACS groups, an unpaired comparison was performed. One sample (OS) comparison of the IF in any group toward the uniformity reference value of 0.5 was also performed, where a significant difference implied a different distribution of a crystallin in the intervals. Statistical analysis of normally distributed data employed the Student’s t-test for OS location tests against a single value or comparisons between paired samples and Welch’s unequal variances t-test for comparisons between unpaired samples^67^where Cohens’ d and d’ coefficients were reported as effect size. Comparisons of data which was not normally distributed were based on the Wilcoxon signed-rank test for OS or paired comparisons and the Wilcoxon rank sum for unpaired comparisons^67^were the probability of superiority was reported as effect size. For all tests the significance level for the two-sided p-value was set to 0.05. Statistical analysis was performed in MATLAB (MathWorks, Natick, MA), using functions of the Statistics and Machine Learning Toolbox and custom-made functions.

## Supplementary Information

Below is the link to the electronic supplementary material.

Supplementary Material 1

## Data Availability

The datasets used and/or analyzed during the current study are available from the corresponding author upon reasonable request.
