# Supplementary material for: Quantitative spatial analysis of crystallin proteins in human lens epithelial cells
Source: Sci Rep. 2025 Oct 3;15:34552. doi: 10.1038/s41598-025-17896-0 (PMC12495019; doi:10.1038/s41598-025-17896-0)
Supplement: Supplementary file 1 — Supplementary Material 1 [file 41598_2025_17896_MOESM1_ESM.docx]

**Supplementary Material**

| Compared signals | | PCC | p_PCC_ | SRCC | p_SRCC_ |
| --- | --- | --- | --- | --- | --- |
| Pixelwise | DAPI vs CRYAB | -0.231 (0.138) | 6.0 10^-25^ | -0.317 (0.118) | 5.5 10^-30^ |
|  | DAPI vs CRYBB2 | -0.314 (0.151) | 3.4 10^-24^ | -0.322 (0.142) | 4.7 10^-26^ |
|  | CRYAB vs CRYBB2 | 0.542 (0.139) | 4.0 10^-39^ | 0.673 (0.110) | 7.7 10^-51^ |
|  | CRYAB vs CRYBB2 cooccurrence region | 0.407 (0.187) | 3.3 10^-25^ | - | - |
|  | CRYAB vs CRYBB2 cytoplasm | 0.501 (0.159) | 7.5 10^-34^ | 0.603 (0.155) | 3.9 10^-39^ |
|  | CRYAB vs CRYBB2 nucleoplasm | 0.554 (0.145) | 9.2 10^-39^ | 0.648 (0.140) | 1.3 10^-43^ |
| Compartment based | CRYAB vs CRYBB2 all compartments | 0.650 (0.149) | 4.3 10^-42^ | 0.805 (0.080) | 3.8 10^-64^ |
|  | CRYAB vs CRYBB2 cytoplasm | 0.568 (0.192) | 1.9 10^-32^ | 0.688 (0.166) | 8.9 10^-41^ |
|  | CRYAB vs CRYBB2 nucleoplasm | 0.646 (0.157) | 1.3 10^-40^ | 0.748 (0.156) | 1.5 10^-44^ |
|  | CRYAB cytoplasm vs nucleoplasm | 0.814 (0.069) | 3.1 10^-68^ | 0.847 (0.093) | 2.6 10^-61^ |
|  | CRYBB2 cytoplasm vs nucleoplasm | 0.749 (0.246) | 4.5 10^-33^ | 0.828 (0.167) | 1.1 10^-45^ |
|  | CRYAB cytoplasm vs CRYBB2 nucleoplasm | 0.649 (0.146) | 1.5 10^-42^ | 0.745 (0.136) | 5.3 10^-48^ |
|  | CRYAB nucleoplasm vs CRYBB2 cytoplasm | 0.499 (0.195) | 6.6 10^-29^ | 0.623 (0.178) | 1.8 10^-36^ |
|  | CRYAB cytoplasm vs CRYBB2 nuclear fraction | 0.465 (0.178) | 2.6 10^-29^ | 0.577 (0.208) | 1.5 10^-30^ |

**Supplementary Table 1**. Correlation coefficients between pixel-wise and compartment-based signals. Mean (SD) over 63 samples.
